# The Association of Hepatitis C Serological Status with Several Risk Factors in Indonesia

**DOI:** 10.1155/2016/3018135

**Published:** 2016-11-20

**Authors:** Noer Endah Pracoyo, Made Ayu Lely Suratri, Roselinda Roselinda, Vivi Setiawaty

**Affiliations:** ^1^Research and Development Center of Public Health Affairs, NIHRD, Jakarta, Indonesia; ^2^Research and Development Center of Health Resources and Services, NIHRD, Jakarta, Indonesia; ^3^Research and Development Center of Biomedical and Basic Technology of Health, NIHRD, Jakarta, Indonesia

## Abstract

Hepatitis is an inflammation of the liver commonly caused by viral infection such as hepatitis A, B, C, D, and E but it is also possible by other causes. Infection with hepatitis C virus is also referred to as a disguise because the early infection is often asymptomatic that often goes undetected. This study aims at determining the several associated risk factors with hepatitis C serological status. The study design is cross-sectional. The biomedical data collection was carried out in 33 provinces in Indonesia with a population in urban blocks, census in Indonesia, where the sample is all household members over the age of one year from selected households by signing the informed consent. Total block census in selected urban area is about 971-block census with a total sample of 15.536 households. The results showed that there is a correlation between hepatitis C serological status and demographic group and that the age and occupation groups showed significant *P* value obtained at 0.001 (OR = 3.27, CI = 1.84–5.81) and 0.209 (OR = 0.23, CI = 0.59–0.94). In conclusion, there are risk factors such as age and occupation that have a correlation of being infected with hepatitis C serological status.

## 1. Introduction

Hepatitis is an inflammation of the liver that can be caused by bacteria, viruses, autoimmune processes, drugs, fatty liver, alcohol, and other harmful substances. The most potential cause of hepatitis is an infection caused by viral hepatitis A, B, C, D, and E. Hepatitis virus is the cause of burden disease and death and it has potential to become an outbreak and a high risk of disease transmission. Hepatitis C typically only shows light such as tiredness, fever, nausea or poor appetite, muscle aches, joint pain, and pain in the liver area. Hepatitis C infection is also known as the silent infection since, in early infection, it is often asymptomatic with no typical symptoms and so it is often overlooked. This condition may continuously progress into an extensive liver damage, which is known as cirrhosis of liver. In some cases, cirrhosis will develop into liver failure, including liver cancer. Most people do not know that they are infected with hepatitis C till the liver damage appears, or through blood tests in the laboratory [1, 2].

At the prior infection, only about 25% of patients show acute hepatitis symptoms, such as weakness, muscle pain, loss of appetite, and fever or sometimes it appears jaundiced on skin and eyes (jaundice), about 75% of patients are without symptoms, even the disease becomes chronic, and often the patient remained asymptomatic (without symptoms). Approximately, 10–15 percent of patients will heal themselves without treatment. This infection is commonly suffered by young women rather than other groups [3–5].

It is estimated that about 50% of hepatitis C cases is transmitted through the use of drugs that are injected [5]. Some cases of hepatitis C virus in Europe were discovered during blood screening transfusion (blood donors); in United States of America, a case was found through current blood screening [5–7]. People who accidentally get needle traces of HCV patients potentially had 1.8% chance of hepatitis C infection [5, 6]. The risk is higher if needles used are hollow and it punctures the wound [5]. About 170 million people are infected with hepatitis C, and nearly 85% of those infected will become chronic, and one-third will develop into cirrhosis or severe damage to liver tissue [5, 6].

About 5–50% infected people in the United States and Canada know their status. The examination of hepatitis C is recommended for people at high risk, including those who have tattoos [8]. Screening is also recommended for people with high levels of liver enzymes since this is often the only sign of chronic hepatitis [9]. Continuous screening is recommended in the United States [6].

The diagnosis of hepatitis C is found if there is HCV RNA in the serum of patients. The tests are very expensive. Therefore, the examination of anti-HCV by ELISA method can be used to screen patients whom are suspected of having hepatitis C, due to its low costs. If the result of anti-HCV shows positive, then it needs to be confirmed by HCV RNA examination. The HCV genotype examination should also be carried out for all patients who will get antiviral therapy to assess the duration of treatment required and the expected response to therapy [7].

Both hepatitis C and hepatitis B are similarly transmitted through blood. In 2007, Ministry of Health Republic of Indonesia conducted National Basic Health Research. The data was collected using a structured questionnaire and also laboratory examination for several diseases including antibody testing of hepatitis B and C. The aim of this study is to determine the association of several risk factors with the serological status of hepatitis C. The analysis of risk factors such as age, gender, medical history, injury/accident history, dental care, occupation, thalassemia, hemophilia, and blood transfusion was conducted based on a questionnaire and linked with the data of anti-hepatitis C serological status.

## 2. Method

In 2007, National Institute of Health Research and Development (NIHRD), Ministry of Health Republic of Indonesia, had conducted the first National Basic Health Research, a nationwide survey with cross-sectional design. Samples of National Basic Health Research 2007 followed the framework of selected National Survey on Social and Economy in 2007. The National Basic Health Research could describe the health profile of the district/city or province level. From each district/city included in the sample frame district/city taken, a number of census blocks were proportional to the number of households in the district/city. A census block included in the sample census block on a district/city is proportional to the number of households in a district/city (probability proportional to size). Overall, a sample census block of the National Survey on Social and Economy 2007 was selected from 17,357 census block samples. In 2007, the National Basic Health Research was able to visit 17,150 census blocks of 438 districts/municipalities. In addition, beside the public health data collection, the survey was also gathering the biomedical data with specimens collection and laboratory testing [8].

Biomedical data was conducted in 33 provinces in Indonesia with the population in urban census blocks in Indonesia. Blood sampling was also conducted on all household members over the age of one year from selected households with a signed informed consent. The series of biomedical sampling selected about 15% of total urban census block of National Survey on Social and Economy. The number of selected census blocks in urban areas reached 971 blocks selected with a total sample of 15,536 households. Blood sampling was not conducted on household members who have had bleeding background regularly.

### 2.1. Statistical Analysis

Statistical analysis was performed with SPSS 15 software. Chi-square test was applied for analysis of categorical data. A *P* < 0.05 was taken as significant for interpretation.

## 3. Results

In Table 1, it indicates that the demographic age group has a significant correlation with anti-hepatitis C serological status at *P* = 0.001 by Odds Ratio (OR) = 3.27 and 95% Confidence Interval (CI) = 1.84–5.81. Occupation shows a correlation with hepatitis C serological status.

Table 2 shows that, in the group medical records, respondents who had dental treatment, thalassemia, hemophilia, and blood transfusion do not have a correlation with hepatitis C serological status.

## 4. Discussion

Hepatitis C is an infection which attacks the liver. One of the causes of hepatitis C is the hepatitis C virus (HCV). Hepatitis C is often asymptomatic, but chronic infection can cause scarring of the liver and lead to cirrhosis. In some cases, people who have cirrhosis also suffer liver failure, liver cancer, or swollen veins in the esophagus and stomach, which can cause bleeding into death [9]. Anti-HCV serological status is positive if the blood level ≥10 IU/mL and it is negative if the blood levels <10 IU/mL. If our body is infected by the virus, it will create anti-HCV antibody towards the hepatitis C virus, which starts from incubation periods and reaches anti-HCV titer ≥10 IU/mL of serological status which stay in 6 months [9].

Hepatitis C showed acute symptoms only in 15% cases [10]. These infections can heal themselves without treatment in 10–50% of patients and are commonly occurring in young women when compared to other groups [7]. In this study, there were no significant differences between women and men with anti-HCV serological status, although there are many women with anti-HCV.

Eighty percent of persons exposed to hepatitis C will develop chronic infection [9]. Most persons who are infected show minimal symptoms or no symptoms at all during the first ten years of infection; hepatitis C causes cirrhosis and liver cancer for people who have been infected [10]. Approximately 10–30% of infected people for more than 30 years will have cirrhosis [6, 10]. Cirrhosis commonly occur to people who are also infected with hepatitis B or HIV and alcoholics [6]. A person with cirrhosis has more risk about twenty times of developing liver cancer, as much as 1–3% per year [6, 10]. Cirrhosis of the liver can cause high blood pressure in the veins which drain the liver, accumulation of fluid in the abdomen, easy bruising or bleeding, dilated veins, especially in the stomach and esophagus, jaundice (yellowing of skin), and brain damage [11]. Based on health data from National Basic Health Research 2007, in the age group, between 15 and 44 years, it was reported that liver disease was on the top of death causes in rural areas, while in urban areas it is in the third rank [8].

Hepatitis C is transmitted through blood and medical equipment contaminated with hepatitis C. According to previous numerous literature, several transmission processes of hepatitis C in the world are caused by the use of injecting drug (60%), through sexual relations (15%), with blood transfusions before screening making up about 10% and the use of means of hemodialysis and perinatal care occupying about 5%. Hepatitis C in developed countries is transmitted through injecting drug use (IDU), whereas, in developing countries, it is transmitted through blood transfusions and unsafe medical equipment [12]. According to the findings from Nelson et al. there were 77 countries that reported prevalence of anti-hepatitis C among injecting drug users and 25 out of 77 countries found the prevalence of anti-hepatitis C to be around 60–80%. In China, USA and Russia, it is reported that it infects about 10 million drugs injecting users; there are about 1.6 million, 1.5 million, and 1.6 million people that suffer from hepatitis C in China, the United States, and Russia, respectively. While the research was conducted at a prison house in the United States, drugs injecting users and tattooing with unsterilized equipment have the potential of being infected with hepatitis C about 10 to 20 times higher than in the common population. Egypt has the highest rate of people who suffer from hepatitis C. The transmission of hepatitis C in Egypt is through the reuse of needles, syringes, vials of drugs that have been used, infuse bags, and unsterilized surgical equipment in the public health facilities and dental care. This risk can be easily removed with standard precautions action [12, 13]. In a study in Iraq, it is reported that the extraction of teeth is a risk factor for developing HCV; the cleaning equipment and sterilization are needed [14]. A dentist belongs to a high risk group of suffering hepatitis. The prevention of hepatitis is conducted by controlling the infection well, in order to reduce the risk of hepatitis by doing sterilization with prophylaxis and vaccination [15].

In this study, we also found a correlation between the variables of people who have oral and dental care at a health facility in the last 12 months; they are going to obtain results with no significance with *P* = 0.050 and risk factors (OR) about 2,26 and 95% Confidence Interval (CI) about 0.98–5.2. Like the research conducted in Egypt, hepatitis C in this country is transmitted through unsterilized surgical equipment in dental and general health services [15]. Likewise, in analysis result of age variable, the respondents aged 5 years have a meaningful relation with the serological status of hepatitis C with the analysis result OR of 0.26 and *P* = 0.001. Since the Odd Ratio result is less than number one, then the relation is not a risk factor but protective one. While on a variable, respondents who do not work have relation with serological status hepatitis C with the result and OR = 0.23, 95% CI (0.594 to 0.129), and *P* = 0.209. The effect is protective because the OR result is less than one. Works related to medical devices contaminated with hepatitis C virus can transmit to workers related to the equipment. Hospital equipment can also transmit hepatitis C virus, including the use of needles and syringes, drugs vials that are continuously used, infuse bags, and unsterilized surgical equipment [15, 16].

## 5. Conclusion

Several risk factors such as dental treatment, thalassemia, and blood transfusion had no correlation of being infected with hepatitis C. The age group 5 years has a high risk to be infected with hepatitis C.

## Figures and Tables

**Table 1:** Relations among the risk factors (ages, gender, and occupation) towards Hepatitis C IgG on National Basic Health Research 2007.

Demographic variable	Serological status (IgG)		OR	95% CI	*P*
	Negative (%) *N* = 21913	Positive (%) *N* = 486			
*Age*					
1–5 years old	1675 (99.3)	12 (0.7)	3.27	(1.84–5.81)	**0.001**
>5 years old	20238 (97.7)	474 (2.3)			

*Gender*					
Male	10234 (98.2)	190 (1.8)	1.9	(0.74–2.86)	0.267
Female	11722 (97.5)	296 (2.5)			

*Occupation *					
Unemployed	2134 (97.4)	58 (2.6)	0.23	(0.59–0.94)	**0.209**
Employed	16382 (97.6)	405 (2.4)			

**Table 2:** Relations among the risk factors of medical records related to IgG (National Basic Health Research 2007).

Medical record variable	Serological status (IgG)		OR	95% CI	*P*
	Negative (%)	Positive (%)			
*(a) Dental treatment*					
(1) Yes	840 (97.7)	20 (2.3)	2.26	(0.98–5.2)	0.050
(2) No	1096 (97.8)	24 (2.2)			
*(b) Thalassemia*					
(1) Yes	31 (100.0)	0 (0.0)	1.02	(1.02–1.03)	0.407
(2) No	21925 (97.8)	486 (2.2)			
*(c) Hemophilia*					
(1) Yes	26 (96.3)	1 (3.7)	0.58	(0.08–4.31)	0.593
(2) No	11190 (97.8)	251 (2.2)			
*(d) Blood transfusion*					
(1) Yes	1881 (98.0)	39 (2.0)	1.15	(0.79–1.66)	0.455
(2) No	4780 (97.7)	114 (2.3)			
